# Childhood tuberculosis—out of the shadows

**DOI:** 10.1186/s41479-016-0022-x

**Published:** 2016-11-24

**Authors:** Ben J. Marais

**Affiliations:** The Children’s Hospital at Westmead and the Marie Bashir Institute for Infectious Diseases and Biosecurity, Sydney Medical School, University of Sydney, Locked Bag 4001, Westmead, Sydney, NSW 2145 Australia

**Keywords:** Tuberculosis, Children, Drug-resistant

Historically, childhood tuberculosis (TB) has been in the shadows, with global TB control programs focused exclusively on adults with sputum smear-positive TB [1]. This was based on a firm belief that the most cost-effective method to contain the TB epidemic and thereby benefit the whole community, including children, was to identify and treat the most infectious adult cases. The fact that this approach excluded children from treatment reflected a strict public health focus and an ignorance of the disease burden suffered by childen in settings with uncontrolled TB transmission. Luckily things have changed. Recently, the United Nations Secretary-General’s Special Envoy on TB Dr Eric Goosby stated: “Far too long, children with tuberculosis have remained in the shadows. While there have been tremendous strides made in improving other areas of child health and survival, we have yet to see the parallel advances in pediatric TB. Instead, many children with TB die before they can be diagnosed and treated” [2].

Key actions to address these challenges are outlined in the “Roadmap for childhood tuberculosis: towards zero deaths”, launched by the World Health Organization (WHO) in 2013 [3]. The Roadmap emphasizes the need for new tools, but also outlines important steps to do better with the policy frameworks and tools already available. It identifies the need for (i) local expertise to guide national TB programs; (ii) pragmatic operational research to address persistent policy-practice gaps; and (iii) advocacy for vulnerable populations without a voice [4]. Children are also firmly embedded in the new WHO End TB Strategy [5], with calls for (i) better data to monitor progress in epidemic control, including drug-resistant TB;(ii) sputum-independent point-of-care diagnostics; (iii) improved treatment options including child-friendly drug formulations; and (iv) new vaccines with enhanced protection against all forms of disease.

In this series, Helen Jenkins presents an overview of the global child TB burden [6]. Of the estimated 1.3 million deaths in children attributed to pneumonia in 2011, most occurred among young children living in TB endemic areas [7]. Child mortality is generally concentrated within disadvantaged communities where the likelihood of TB exposure and infection is greatest. TB is thought to be a major contributor to under-five mortality in these settings, although children dying from TB are often incorrectly classified as pneumonia, meningitis, human immunodeficiency virus (HIV)/acquired immune deficiency sydrome (AIDS) or malnutrition deaths [8]. Jenkins emphasizes the need to make better use of active case-finding and preventive therapy strategies, because TB is a preventable and treatable disease from which no child should die.

Claudia Roya-Pabon and Carlos Perez-Velez consider optimal approaches for diagnosing TB [9]. Accurate TB diagnosis continues to be a challenge in young children, due to a myriad of clinical and radiological presentations that can mimic many common childhood diseases. Bacteriological confirmation of young children with non-cavitating lung disease is most problematic, due to difficult specimen collection and the paucibacillary nature of their disease. The authors present a detailed diagnostic approach that should minimize both under- and over-diagnosis and assist clinicians to manage cases appropriately, even in resource-limited settings.

Helena Rabie and Pierre Goussard review tuberculosis and pneumonia in HIV-infected children [10]. HIV-infected children have a high burden of lower respiratory tract infections and TB is particularly problematic in settings where TB/HIV co-infection is common among adults. Universal early antiretroviral therapy (ART) improves survival and reduces the burden of opportuntistic infections in HIV-infected children, but pathogens such as *Pneumocystis jirovecii* and cytomegalovirus should still be considered, along with TB. There is growing recognition that *in utero* HIV exposure has detrimental immunological effects, even if babies do not acquire HIV infection.

James Seddon and Simon Schaaf describe advances in the treatment of children with TB [11]. New treatment regimens are being evaluated that have the potential to shorten treatment duration in children with minimal disease. An optimally formulated child-friendly, dissolvable, fixed-dose combination tablet (developed by the TB Alliance) has recently been made available via the Global Drug Facility. Rising rates of drug-resistant TB and confirmed epidemic spread of these drug-resistant strains threaten global TB control efforts [12]. Modeling data suggest that around 45,000 children develop drug-resistant TB every year [13]. Unlike adults, treatment outcomes for children with drug-resistant TB are excellent, but few are able to access proper diagnosis and care.

Lastly, Jamie Triccas and Claudio Counoupas review novel vaccination approaches [14]. Given the huge disease burden and diagnostic challenges highlighted, children would benefit greatly from a vaccine that offers enhanced protection against severe childhood TB. In addition, a vaccince that protects against adult-type disease will limit ongoing transmission and be crucial in the fight to end TB. Novel vaccine strategies include recombinant forms of the existing Bacille Calmette-Guerin (BCG) vaccine, protein or viral-vectored vaccines to boost BCG-induced immunity, and live attenuated forms of *Mycobacterium tuberculosis.* There are promising vaccine candidates, but it is difficult to define optimal “real-life” disease end-points, assess potential BCG replacement (given the non-specific all-cause mortality benefit attributed to BCG) and identify the most suitable candidates to progress to expensive clinical trials.

There is a need for better collaboration between pediatricians, national TB control programs and maternal and child health initiatives in TB endemic countries to improve the detection and management of children with TB. Priority actions that have been identified in a consensus call to action [15] include:

- empowering children, their families, and communities to advocate for improved access to TB prevention, diagnosis and care
- stepping up programatic efforts to identify children and adolescents most at risk of TB and prevent, diagnose and treat them with the best diagnostic tools and medicines available
- strengthening health systems at all levels, and integrating, where possible, TB activities with programs focused on maternal and child health, HIV/AIDS, and nutrition
- including children and adolescents in research activities at the earliest possible stage to accelerate the development of appropriate diagnostics and treatments
- scaling up investment in the development of childhood TB diagnostics, treatment and vaccines, as well as the health systems that use them.
